# Higher visual function deficits are independent of visual acuity measures in children with cerebral visual impairment

**DOI:** 10.3389/fnhum.2024.1451257

**Published:** 2024-10-15

**Authors:** A. Chandna, M. Wong, S. Veitzman, E. Menjivar, A. Kulkarni

**Affiliations:** ^1^Smith Kettlewell Eye Research Institute, San Francisco, CA, United States; ^2^Alder Hey Children’s Hospital, Liverpool, United Kingdom; ^3^Computer Science Department, San Francisco State University, San Francisco, CA, United States

**Keywords:** CVI, children, question inventory, higher visual function, higher visual function deficits, VA, semi-structured, interview

## Abstract

Cerebral visual impairment (CVI), the leading cause of bilateral visual impairment in children, is often characterized by visual acuity (VA) loss and higher visual function deficits (HVFDs). However, the relationship between VA loss and HVFDs remains unknown. A previous study using the Higher Visual Function Question Inventory (HVFQI) demonstrated that normal VA did not preclude HVFDs. In this prospective controlled study of children with CVI, we examine the relationship between HVFDs and degrees of VA loss to refine our understanding of this relationship. We introduce two new indices—HVFD spectrum and severity—to provide a comprehensive view of how CVI affects the individual child and the entire cohort. We also performed an analysis to determine the effectiveness of the HVFQI in eliciting HVFDs and present a preliminary analysis of the relationship between HVFDs and age. The study participants included 59 children with CVI (age: 9.87 ± 3.93 years [mean ± SD]; binocular VA: 0.35 ± 0.34 log MAR.) and 120 neurotypical (NT) children with normal visual acuity (age: 8.7 ± 2.8 years; binocular VA: 0.14 ± 0.16 logMAR). Clinical history and notes independently confirmed the diagnosis of CVI. Parents were interviewed with the HVFQI, and their responses were recorded using a five-level Likert scale. Mann–Whitney U-test (MWU) determined the ability of HVFQI to distinguish between CVI and NT participants; Fisher’s exact test (FET) and d-variable Hilbert–Schmidt independence criteria (dHSIC) assessed the independence between HVFDs and VA. The average spectrum (range 0–1) and severity (range 1–5) indices for CVI (spectrum: 0.65 ± 0.24, severity: 3.1 ± 0.77) and NT (spectrum: 0.12 ± 0.17, severity: 1.42 ± 0.49) were markedly different. MWU (*p*-value <0.00001) confirmed the ability of HVFQI to distinguish CVI from NT children for both indices. The FET reported a *p*-value of 0.202, which indicates that the data does not exhibit any relation between the HVFDs severity and VA. Analysis using dHSIC supports these findings (*p*-value 0.784). Based on these results, we urge that all children with suspected CVI need to be assessed for HVFDs in addition to VA measures. The HVFQI can potentially increase our understanding of the neural basis of visual perception, cognition, and visually guided action and lead us toward a conceptual model of CVI, translating to clinical practice improvements.

## 1 Introduction

Measures of visual acuity (VA) and assessment for higher visual function deficits (HVFDs) are critical components for the diagnosis and management of cerebral visual impairment (CVI). VA, a common measure of a “visual function” (i.e., of how the eye functions), forms the basis for defining visual loss and disability from ocular disease and, by extension, visual impairment from brain-based visual loss, which affects how the individual functions in their environment or “functional vision” (Colenbrander, 2003; Bennett et al., 2019). CVI, a brain-based condition, leads to challenges with functional vision. VA measures are limited to the narrow assessment of one aspect of visual function (i.e., spatial resolution) in conditions of high luminance and contrast but inadequate for a comprehensive assessment of other visual functions (e.g., contrast sensitivity). Importantly VA measures do not assess how vision is utilized in everyday visual activities mediated through higher visual functions (HVFs). While terminology in the field is variable, for example, higher visual functions (Rolls, 1991) and higher-level visual perception (Itzhak et al., 2023), we have used HVFDs (Zihl and Kennard, 2003) to characterize higher visual processing deficits ascribed to the dorsal and ventral streams. Deficits in processing within the inner retina and optic nerve are typically classified as ocular problems, whereas brain-related visual processing deficits fall under the category of HVFDs (Colenbrander, 2010a; Ortibus et al., 2011, 2019; Bennett et al., 2019). For determining HVFDs, an assessment of HVFs or visually guided behaviors is necessary (Dutton and Jacobson, 2001; Bennett et al., 2019; Ortibus et al., 2019). VA and HVFs are both affected in CVI and significant HVFDs may be present in presence of normal VA and vice versa (Chandna et al., 2021; van Genderen et al., 2012; Bosch et al., 2014). Historically, CVI was diagnosed on the basis of VA alone (Marquis, 1934; Whiting et al., 1985). However, a review of literature regarding the terminology and manifestations of CVI (Hoyt, 2003; Colenbrander, 2010a; Jacobsen, 2014; McConnell et al., 2021) and the development of a consensus definition of CVI (Sakki et al., 2017) strongly suggests the inclusion of assessment of HVFDs in the management and the need for this change to be explicitly reflected in the international classification systems of visual impairment (Colenbrander, 2010b; Ravenscroft, 2016; Sakki et al., 2017; Kran et al., 2019).

HVFs can be explained through a functional model of two distinct but interconnected cerebral networks; the dorsal stream connecting occipital and largely posterior parietal lobes (occipital-parietal pathway) (Gallivan and Goodale, 2018; Weiller et al., 2021) and the ventral stream connecting the occipital and inferotemporal cortical area (occipital-temporal pathway) (Weiller et al., 2021). The dorsal stream executes visually guided action, which is termed the pathway for action. The ventral stream aids in the recognition of shapes, objects, and faces, which is termed the pathway for cognition (Milner, 2017; Goodale et al., 2005). There are anatomical interconnections between the dorsal and ventral stream such as the vertical occipital fasciculus (Yeatman et al., 2014; Bauer and Merabet, 2024), which serve to integrate higher visual functions such as reach and grasp action. Additional white matter pathways to cortical areas such as frontal lobe (Ortibus et al., 2012; Bauer and Merabet, 2024; Sarubbo et al., 2013; Dutton et al., 2004) and sub-cortical areas (Merabet et al., 2017), especially superior colliculus (Sprague and Meikle, 1965), contribute to multisensory integration (Chokron and Dutton, 2016), visuomotor execution (Polanen and Davare, 2015), and eye movement control (Chang and Borchert, 2021) to complete visual perception and action.

From this model, it is possible to build a hypothesis that the loss of VA and HVFs may be affected selectively or together but not be interdependent. Though van Genderen et al. (2012) and Chandna et al. (2021) reported HVFDs in the presence of near-normal VA in children with CVI, they did not study the relationship between HVFDs and different levels of VA loss in CVI. VA measures and HVFDs have been reported as part of a study, but the relationship has not been directly examined (Morelli et al., 2022) and the specific VA results were not correlated with the results of the Children’s Visual Impairment Test for 3- to 6-year-olds (CVIT 3–6) to assess visual perceptual functions (Vancleef et al., 2020). This relationship is important for a holistic view of the visual challenges faced by children with CVI (Philip and Dutton, 2014; Bennett et al., 2019; Kran et al., 2019). For the assessment and intervention, it is important to know if HVFDs are independent of VA. Are HVFDs less affected in children with mild to moderate VA loss? Is the spectrum and severity of HVFDs related to VA measures? Does age matter? The answers to these questions would provide an understanding of the visual capabilities of the child and (re)habilitation measures needed to support the child, their family, the teachers, and the school. It may provide insight into the division and the interrelationship between conscious vision (visual cognition; VA; the ventral stream) and non-conscious vision (dorsal stream, action guided by vision without conscious visual perception or awareness) (Philip and Dutton, 2014) and the possible reasons for the relationship between VA measures and HVFDs.

Previously, children with CVI and normal VA were studied (Chandna et al., 2021) where we reported that normal VA did not preclude HVFDs. In this study, we examine the relationship between HVFDs and different levels of VA loss in CVI to extend the relationship between VA land HVFDs, discuss the implications of the results for CVI, and increase our understanding of visual perception, cognition, and visually guided action.

## 2 Materials and methods

This study received the Institutional Review Board approval (CHA002) and abided by the principles of the Declaration of Helsinki. Informed written consent was obtained from parents and assent from children where appropriate.

### 2.1 Higher visual function question inventory (HVFQI)

The HVFQI, a clinical assessment instrument, elicits answers to questions on visual behaviors associated with higher visual function deficits (visual perceptual deficits) in CVI. The original clustering of questions into putative domains was retained from the original CVI-I (Dutton and Bax, 2010; Macintyre-Béon et al., 2010, 2012; Ortibus et al., 2011; Philip et al., 2016). The HVFQI had been modified as described in Chandna et al. (2021) from CVI-I (Dutton et al., 2010; Macintyre-Béon et al., 2012). An additional seven questions were added to this HVFQI following a recent review of literature and discussion with Professor Gordon Dutton. However, for an accurate comparison and analysis, we report on the same 51 questions that were included in Chandna et al. (2021). The responses were tabulated along a Likert scale. Participants chose from *Never*, *Rarely*, *Sometimes*, *Often*, and *Always*. A *Not Applicable* option was included since several of the observed behavior items may not be developmentally appropriate or not assessed due to comorbid conditions. *Not Applicable* responses were accounted for in the analysis as described in Chandna et al. (2021). The HVFQI was delivered through remote video interviews with a web-based application—the HVFQI web app.

### 2.2 Recruitment

Study participants were recruited through clinical institutions, educational networks, CVI support groups, information websites and social media groups, parents, teachers of the visually impaired, colleagues, and friends. Following an expression of interest in the research project by the parent or caregiver, the research information and consent and assent documents were provided for their review. A face-to-face virtual meeting (Zoom) was set up across time zones at a mutually convenient time, At the meeting, informed digital e-consent and consent for sharing of clinical information was obtained. Assent, with parental involvement, was obtained for children over 12 years of age, if present at the interview and cognitively appropriate for the child.

### 2.3 HVFQI interview

The HVFQI interview via Zoom was commenced with standardized instructions. A unique participant ID was generated. Single, randomly generated questions were displayed on a shared screen to the participant along with the range of possible responses (the Likert scale and the *Not Applicable* options). The parent was encouraged to read the question and choose one of the options. If requested, questions were clarified with non-leading explanations. Comments from the parent were noted in the free text section on the screen. If the *Not Applicable* option was chosen, the reason was documented. The HVFQI was completed in one session and within-session breaks were given if necessary. We did not control the response time. While the neurotypical and CVI cohorts were not timed for (Chandna et al., 2021), responses collected using the HVFQI web app were annotated with timestamps. The range of time taken for all respondents via the app (CVI and NT) varied between 6 and 67 min (mean ± SD: 31.15 ± 13.45 min, n = 61). The average questionnaire response time for CVI participants took twice as long (33.60 ± 13.17 min, n_CVI_ = 52) than for neurotypical (NT^1^) participants (15.10 ± 13.32 min, n_NT_ = 9).

### 2.4 Participants

This study introduced new participants, both neurotypical and those with a CVI diagnosis. The overall analysis included previously reported participants from Chandna et al. (2021) (see Table 1).

**Table 1:** Combined participant statistics from current study and 2021 study.

Group	Size (*n*)	Age (years) Mean ± SD	VA logMAR Mean ± SD	Gender distribution (F/M/Unknown)
CVI	92	9.4 ± 3.6 yrs.	0.26 ± 0.29	45.7% / 54.3% / 0.0%
Typical	120	8.7 ± 2.8 yrs.	0.14 ± 0.16	43.3% / 54.2% / 2.5%
Overall	212	9.2 ± 3.4 yrs.	0.11 ± 0.23	44.3% / 54.2% / 1.4%

#### 2.4.1 Children with CVI group

A total of 59 children (35 boys, 24 girls) with an established diagnosis of cerebral visual impairment (CVI) participated in the study. The history and clinical details of the participating children were obtained through a direct semi-structured interview and documented on our standard form. We recorded a detailed history and present status of vision, details of onset and course of CVI, comorbid conditions, course of pregnancy, birth history, adverse events, and developmental and family history. Clinical documents and reports of investigations including brain imaging were obtained. The diagnosis of CVI, established prior to participation in this study, was confirmed as part of the inclusion process.

The mean age of the participants was 9.95 ± 3.95 years. The mean binocular VA was 0.26 ± 0.29 Log MAR. Eight children (13%) had acuity worse than 1.0 Log MAR. Out of the participating children, 36 (60%) were born at term; three born post-term; and 21 (40%) were born preterm (pregnancy term: one, early; 12, moderate; 4, very preterm; and 4, extremely preterm; World Health Organization classification). The causes of CVI were related to brain injury ranging from cerebral hemorrhage (20; 33%), meningitis (8; 13%), cerebral hypoxia (6; 10%), brain malformation (4; 7%), metabolic disturbance (3; 5%), head trauma (1; 2%), brain tumor (1; 2%,) and genetic causes (17; 28%). There were multiple comorbid conditions such as autism (25; 42%); attention-deficit hyperactivity disorder (ADHD) (10, 17%), and central auditory processing deficit (CAPD) (5, 8%). Some children had more than one cause and comorbid condition.

#### 2.4.2 Additional CVI cohort from 2021 study

For comparison, we included the previous cohort of 33 children with CVI (mean age ± SD: 7.0 ± 2.8 years) and with those near normal VA (0.14 ± 0.12 logMAR) (Chandna et al., 2021). Exploratory data analysis did not reveal any significant differences (except VA) between the 2021 and the present cohort. Therefore, the two cohorts were merged to form the definitive cohort (Table 1). The results in the main body of this article are presented as a combined group.

#### 2.4.3 Neurotypical group includes cohort from 2021 study

The majority of the neurotypical group (NT) of 111 typically developing children (mean age 8.7 ± 2.8 years and VA of 0.14 ± 0.16 logMAR) were interviewed in (Chandna et al., 2021). An additional nine neurotypical children (3 boys, 6 girls; mean age 9.7 ± 3.4 years) with normal VA responded to the question inventory for this study, extending the reference sample size. All children were assessed with the same detailed medical and vision history (including a detailed interview regarding present and past eye and general health; pregnancy and birth history; past medical and surgical illnesses; family history of eye and general health, especially inherited conditions; a social history; current medication list and results of all available medical tests and investigations) and HVFQI (Chandna et al., 2021; the additional seven questions in the recent HVFQI were not included in the analysis). An initial exploratory comparative analysis of the two groups did not reveal any significant differences. The two groups were combined and the results are presented as a combined group (Table 1).

#### 2.4.4 Combined groups

The combined cohort (2021 and the present cohort) comprised 92 CVI participants and 120 NTs (see Table 2).

**Table 2:** Participants’ CVI causes and comorbidities.

Cause	Size (*n*)	Relative size
Cerebral hemorrhage	20	33%
Meningitis	8	13%
Cerebral hypoxia	6	10%
Brain malformation	4	7%
Metabolic disturbance	3	5%
Head trauma	1	2%
Brain tumor	1	2%
Genetic causes	17	28%

Comorbidities	Size (*n*)	Relative size
Autism	25	42%
ADHD	10	17%
CAPD (Central Auditory Processing Disorder)	5	8%

### 2.5 Data collection and storage

All clinical data was transcribed to the database. Non-identifiable HVFQI data collected with the help of the HVFQI web app from each interview was downloaded to a dedicated database. All data was stored on a secure site. Access to the data is granted through authentication credentials and a simple role-based access control security model; all communication to the server was encrypted.

## 3 Data analysis: objectives, methodology, and results

In this section, we present a three-part data analysis. First, we (re)validate the HVFQI with the new cohort of children with CVI with several levels of VA loss building on our previous study where we validated the HVFQI for children with normal VA in comparison with NTs (Section 3.1). Second, we examine the relationship between HVFD and VA (Section 3.2). Finally, we present our early analysis of HVFDs with age (Section 3.3). For the HVFQI analysis, we analyzed the same 51-question HVFQI (Chandna et al., 2021); the additional nine questions in the recent HVFQI were not included in the analysis.

### 3.1 Objective 1: evaluate the efficacy of the HVFQI questionnaire in identifying and characterizing higher visual function deficits in children with CVI and its ability to distinguish neurotypical children

Tools that are effective at characterizing the HVFDs and that are inexpensive and easily available anywhere in world are essential to achieve impactful and sustained advancements in CVI research and practice. The study by Chandna et al. (2021) introduced one such tool, the HVFQI questionnaire, which was shown to be effective at determining HVFDs. Importantly, Chandna et al. (2021) also demonstrated that the information gathered by the HVFQI questionnaire was effective at identifying children with CVI from neurotypicals. In the current study, we seek to conclusively establish the effectiveness of the HVFQI questionnaire using a substantially larger participant pool. We also introduce two new metrics that quantify the spectrum and severity of HVFDs to provide a two-dimensional view of this complex visual impairment.

#### 3.1.1 Severity of HVFDs

##### 3.1.1.1 Methodology

For this analysis, we replicate the experimental methodology employed by Chandna et al. (2021). Specifically, the two participant groups being studied (CVI and NT) are compared using the HVFD Severity Index.^2^ The HVFD Severity Index is computed for each participant and is defined as the arithmetic mean of the participant’s responses to the HVFQI. To operationalize this, every categorical response label is mapped to a numeric value.^3^ *Never* maps to score 1, *Rarely* to 2, *Sometimes* to 3, *Often* to 4, and *Always* to 5. *Not Applicable* responses are filtered out (see Chandna et al., 2021 for methodology). The HVFD Severity Index is bounded between 1 and 5, with both values inclusive. Once the Severity Index is computed for every participant in the group, the distribution of Severity Indices for CVI group is compared with the distribution of the Severity Index for the NT group. The two distributions are statistically compared using the Mann–Whitney U-test and rendered as box-and-whisker plots for visual comparison.^4^

##### 3.1.1.2 Results

**Figure 1:**
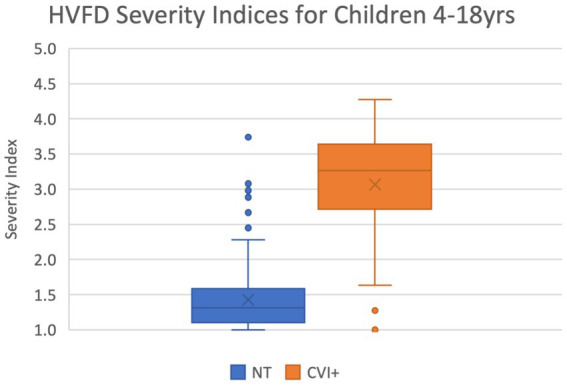
Box-and-whiskers representation of the distribution of HVFD Severity Index for group NT (4- to 18-year-old neurotypical children, *n* = 120) and group CVI (4- to 18-year old children with CVI, *n* = 92). Mann–Whitney U-test comparing the two distribution reports *p*-value << 0.00001.

Figure 1 shows the box-and-whiskers plots for the HVFD Severity Index distributions in the NT and CVI groups. It is evident from the figure that the two distributions are markedly different; HVFD Severity Indices for neurotypical children are substantially lower than those of the children with CVI. The Mann–Whitney U-test comparing the two distributions provides a *p*-value <0.00001, thus, confirming the difference in the HVFD Severity Index distributions of the two groups as statistically significant.

The median HVFD Severity Index for the NT group is 1.27 while that for the CVI group is 3.23, illustrating that the children with CVI have noticeably more severe HVFDs than neurotypical children. The other two quartiles (25 and 75% of the group) exhibit similar trends where the quartile values of HVFD Severity Index for the CVI group is substantially higher than that of the NT group.

#### 3.1.2 Spectrum of HVFDs

##### 3.1.2.1 Methodology

To study the breadth of the deficits that a participant reports, we introduce a new metric: HVFD Spectrum Index, which is defined as the ratio of the number of responses with *Sometimes* or higher response category (i.e., *Sometimes*, *Often*, *Always*) and the total number of responses for that participant. For example, a participant who has responded to 30 questions on the HVFQI with *Sometimes*, *Often*, or *Always*, and to the remaining 21 questions with *Never* or *Rarely*, the HVFD Spectrum Index will be 30/51 = 0.588. The HVFD Spectrum Index is bounded between 0 and 1. The NA responses were accounted for (Chandna et al., 2021).

Using this new metric, we compare the two study groups by first computing the HVFD Spectrum Index for every participant in the two groups, and then computing the five-point summary of the resulting distributions of HVFD Spectrum Indices for NT and CVI groups using box-and-whiskers plots.

##### 3.1.2.2 Results

**Figure 2:**
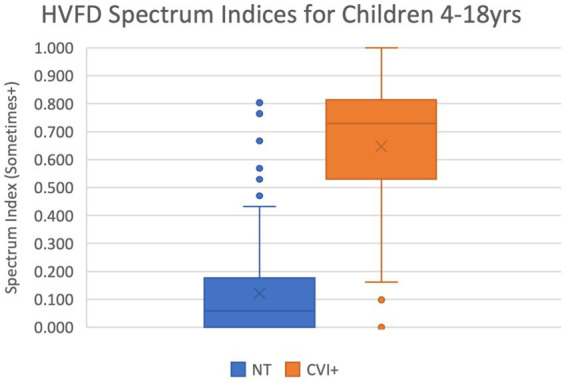
Box-and-whiskers representation of the distribution of HVFD Spectrum Index for group NT (4- to 18-year-old neurotypical children, *n* = 120) and group CVI (4- to 18-year-old children with CVI, *n* = 92). Mann–Whitney U-test comparing the two distribution reports *p*-value << 0.00001.

Figure 2 shows the corresponding box-and-whiskers plots. The distribution of the HVFD Spectrum indices for the NT group is markedly distinct from that of the CVI group, which is confirmed by the Mann–Whitney U-test result with a *p*-value <0.00001. The CVI group was observed to have a much wider spectrum of deficits than the NT group participants. As indicated by the median values, half of the children with CVI are reporting deficits on 74% of the questions, while half of the neurotypical children are reporting deficits on 6% of the questions on the HVFQI. On average, the CVI group is reporting HVFDs for 65% of the questions while the NT group is reporting only them only for 13% of the questions (shown by cross marks in the box plots).

Among the NT group, seven participants reported unusually high HVFD Spectrum Indices ranging from 4.7 to 8.1 (shown in Figure 2 as six points; two datapoints overlap at 8.1). These outliers are the same individuals as previously discussed with high HVFD Severity Indices and may include participants with undiagnosed CVI. The CVI cohort has five participants (four overlapping at 0.0) that report uncharacteristically low HVFD Spectrum Indices; these are the same individuals that responded with low HVFD Severity Indices and the previous justification discussion applies.

#### 3.1.3 Joint analysis of severity and spectrum of HVFDs

##### 3.1.3.1 Methodology

In this analysis, we study the relationship between the spectrum and severity of HVFDs to develop a more comprehensive picture of CVI. We use the simple tool of scatter plot for this analysis where each participant is represented by their HVFD Severity Index (X-axis) and Spectrum Index (Y-axis). The participants from the two groups, CVI and neurotypical, are distinguished on the scatter plot with datapoints displayed in different colors.

##### 3.1.3.2 Results

**Figure 3:**
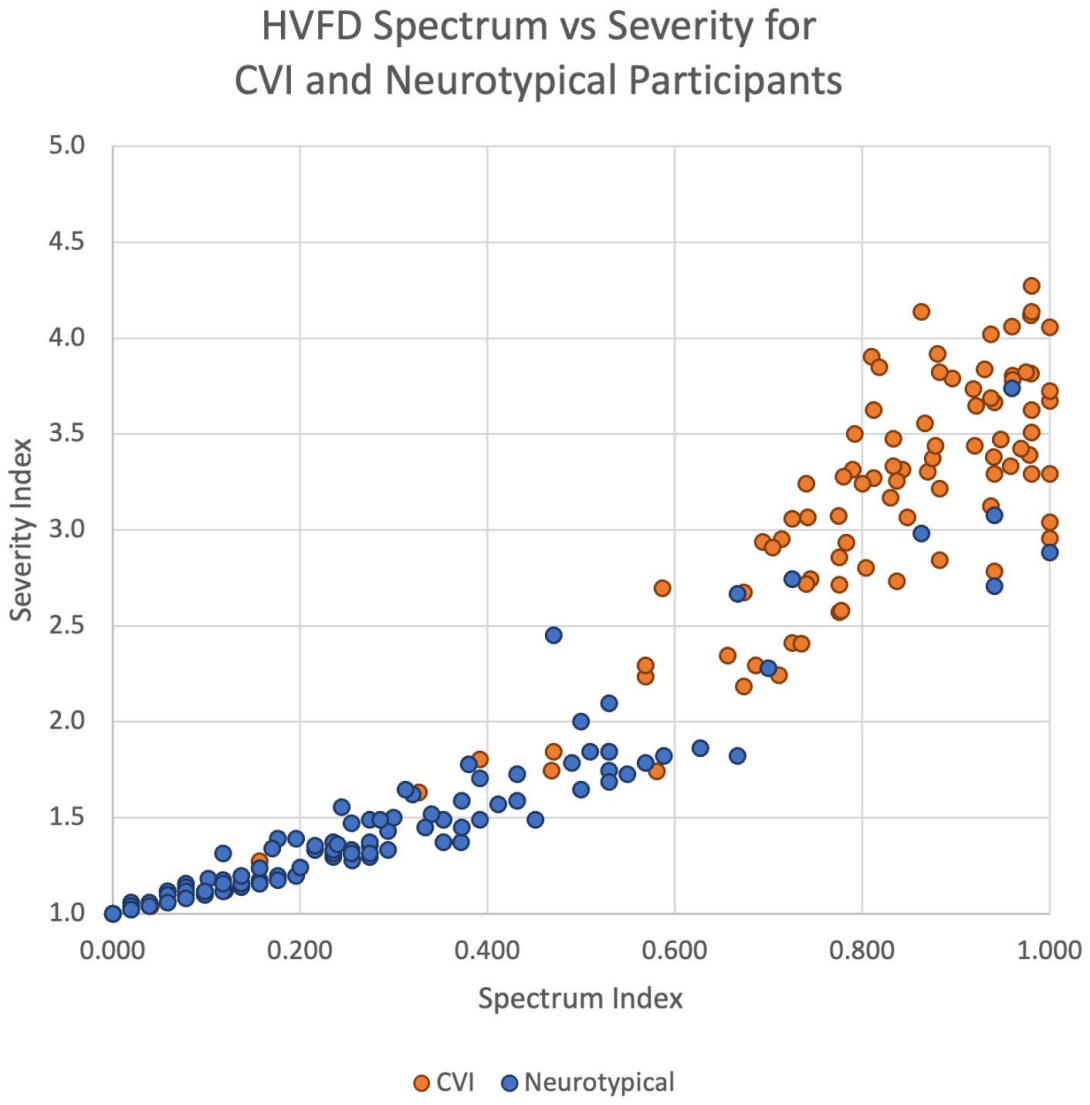
Scatter plot of HVFD Severity Index versus HVFD Spectrum Index for group NT (4- to 18-year old neurotypical children, *n* = 120) and group CVI (4- to 18-year-old children with CVI, *n* = 92).

The scatter plot in Figure 3 shows that the majority of the datapoints that correspond to neurotypical children are concentrated in the lower left section of the plot (low severity and low spectrum indices), while those for CVI children are in the top right section (high severity and high spectrum indices).

It is worth noting that for the NT group (excluding the outliers), the spread along the spectrum dimension is wider (0 to 0.6, which is 60% of the range of spectrum metric) than that along the Severity Index (1.0 to 2.0, which is 25% of the range of severity metric). This again suggests that there may be participants with undiagnosed CVI type of HVFDs in the neurotypical group (Williams et al., 2021).

When comparing the two groups, NT and CVI, in terms of the spread of their indices on both metrics, the consistent trend is that the dispersion is wider for CVI group than for NT group, thus illustrating the extent of variability of symptoms in the CVI group, quantitatively supporting the heterogeneity of visual deficits in CVI, and underscoring the need for thorough HVFD profiling.

The analyses in the previous sections (Sections 3.1.1, 3.1.2, and 3.1.3) conclusively establish that the higher visual functions among children with CVI exhibit distinctly higher severity and wider spectrum of deficits than those among neurotypical children when using HVFQI questionnaire to collect this information.

#### 3.1.4 Distinguishing CVI from NT participants at different severity levels

##### 3.1.4.1 Methodology

This next data analysis investigates if the HVFQI questionnaire can distinguish NTs from CVI with different severities of HVFDs. We adopt the same experimental methodology as Chandna et al. (2021) for this analysis where the CVI group is compared with the neurotypical group at each of the four response levels: *Rarely*, *Sometimes*, *Often*, and *Always*. Specifically, for a given response level, the ratio of the number of questions with responses equal or higher than that level and the total number of questions is computed for each participant. We refer to this ratio as response relative frequency (RRF). For example, RRF@Rarely+ metric for a participant who has responded to 49 out of the 51 questions with *Rarely* or higher response will be 0.960 (49/51); similarly, RRF@Often+ for a participant who has responded to 19 out of 51 questions with *Often* or *Always* would be 0.373 (19/51). The RRF metric is bounded between 0 and 1. We computed the four RRF values, *Rarely*+, *Sometimes*+,^5^ *Often*+, and *Always*, for each participant in both CVI and NT study groups.

##### 3.1.4.2 Results

**Figure 4:**
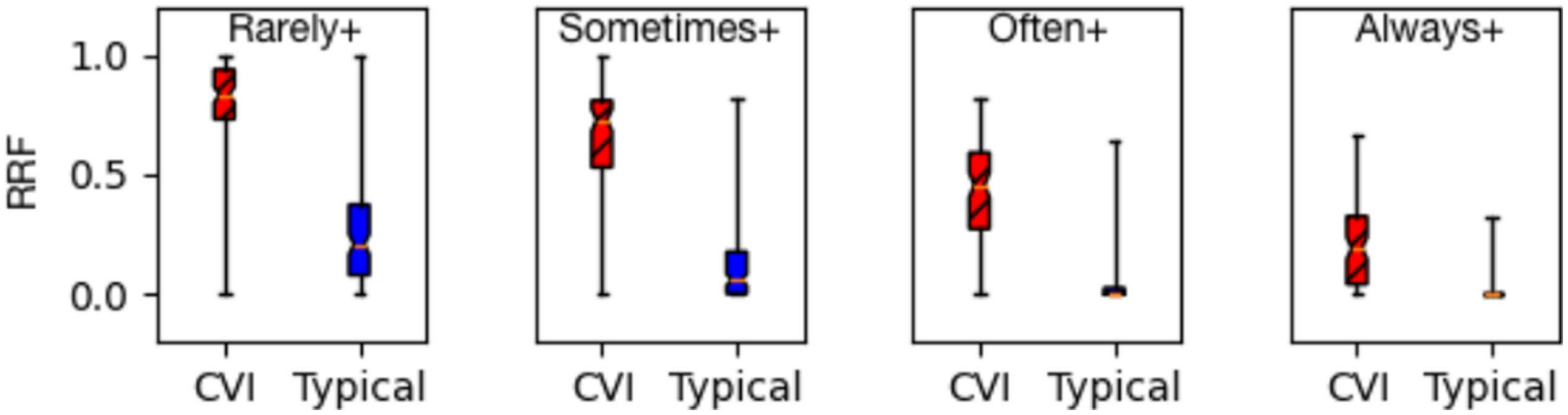
Response relative frequency (RRF) at all dichotomies (Rarely+, Sometimes+, Often+, and Always+) group CVI (children with CVI, *n* = 92) are separate from group typical (neurotypical children, *n* = 120) shown as a box-and-whiskers plot (MWU *p*-value ≪ 0.00001).

Figure 4 shows the box-and-whiskers plots comparing the two resulting distributions for CVI and NT at each response level. The plots were created as described in Chandna et al. (2021), updated with the inclusion of children with visual acuity impairments ranging from mild to blind as seen in Table 3 (the previous study focused on normal visual acuity). We draw the following observations from these results.

1. The effectiveness of the HVFQI questionnaire in distinguishing children with CVI from neurotypical children holds irrespective of the severity of their higher visual function deficits.
2. At each severity level, the RRF values for the CVI group are markedly higher than those for the NT group. The Mann–Whitney U-test formally compares the distributions for all four severity levels and reports *p*-value << 0.00001, thus establishing the statistical significance of the differences.

**Table 3:** World Health Organization (2019) visual acuity categories and CVI participants per category.

Category	Normal	Mild	Moderate	Severe	Blind
Visual acuity (logMAR)	0–0.3	0.3–0.5	0.5–1.0	1.0–1.3	1.3 or higher
CVI participants	53	10	14	3	1

#### 3.1.5 Question-level analysis of HVFD severity

##### 3.1.5.1 Methodology

This analysis zooms in on individual questions to compare the two groups, CVI and NT, in terms of their HVFD severity. For each of the 51 questions on the HVFQI questionnaire, the arithmetic mean (and one standard deviation) of all the responses (severity) by the CVI group participants is computed. The same process is repeated for the participants from the neurotypical group. These question-level arithmetic means and standard deviations for the two groups are then plotted to facilitate comparative analysis.

##### 3.1.5.2 Results

**Figure 5:**
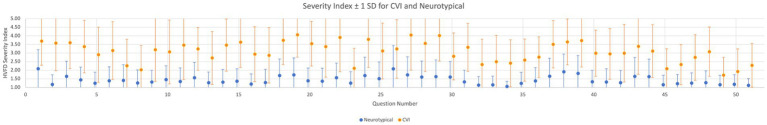
Question-level HVFD severity. Arithmetic mean and one standard deviation of participant responses for each of the 51 questions for group CVI (children with CVI, *n* = 92) and group neurotypical (neurotypical children, *n* = 120). Treating each question independently, mean MWU *p*-value <0.00001 for CVI and NT response distributions.

A consistent trend across all 51 questions is evident in Figure 5 where the response means for the CVI group are distinctly higher than the response means of the neurotypical group. For some questions, for example, Q7, Q8, and Q23,^6^ the standard deviation bars for the two groups show a higher overlap. Our age range was 4–18 years and these responses may be affected by developmental age and indicate the presence of CVI traits in the NT population (Williams et al., 2021).

#### 3.1.6 Question-level analysis of HVFD prevalence

##### 3.1.6.1 Methodology

**Figure 6:**
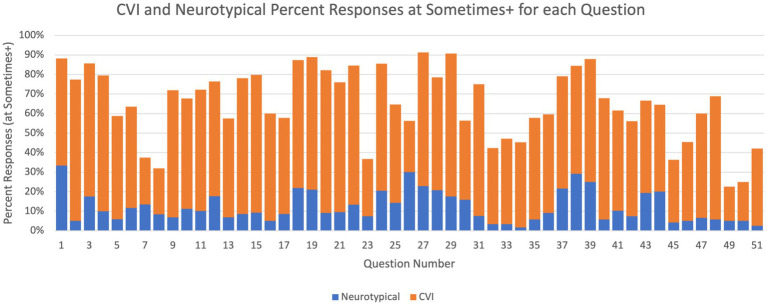
Question-level HVFD prevalence. Percentage of participants with *sometimes* or higher response in the neurotypical group (children with CVI, *n* = 92) and in the CVI group (neurotypical children, *n* = 120).

This analysis also operates at the individual-question level but with a focus on comparing the prevalence of each deficit across the two study groups. Specifically, for each of the 51 questions, the percentage of participants that selected *Sometimes*, *Often*, or *Always* is computed for each group and then compared using a bar plot (see Figure 6).

##### 3.1.6.2 Results

These results also demonstrate stark differences in the trends exhibited by the two study groups. For every question, substantially larger percentage of participants from the CVI group responded with presence of that deficit than participants from the NT group. For Q1, 88% of CVI children responded *Sometimes* or higher, while for neurotypical children that was only 34%. One of the largest differences between the two groups is observed for Q2 (4% for NT vs. 78% for CVI), and the smallest difference for Q49^7^ (4% for NT vs. 22% for CVI).

Overall, the results from these data analyses confirm the efficacy of the HVFQI questionnaire in eliciting, quantifying, and characterizing the higher visual function deficits experienced by children with CVI. Consequentially, these analyses also demonstrate that the HVFQI questionnaire can be used effectively to distinguish children with CVI experiencing HVFDs from neurotypical children.

### 3.2 Objective 2: examine the relationship between visual acuity and HVFDs in children with CVI

Whether and how visual acuity (VA) and HVFDs in children with CVI are related is a long-debated question in the field. So, the findings from an investigation into this question are likely to have a broad impact on CVI diagnosis, research, and practice. VA and HVFD have been directly investigated for normal visual acuity (Chandna et al., 2021) but not for different levels of VA and HVFDs. However, the relationship between visual function (VA) and functional vision (HVFD) has been questioned in low vision (Colenbrander, 2003; Colenbrander, 2010a) and in CVI (Morelli et al., 2022; Kran et al., 2019). A systematic assessment of this relationship is lacking. Here we directly examine whether HVFDs are related to the level of VA loss both for the severity (2a) and the spectrum (2b) of HVFDs.

#### 3.2.1 Data

Out of the 92 children with CVI, the VA measurements were available for 83 children. The remaining nine children could not be assessed on a logMAR VA chart due to cognitive reasons.

#### 3.2.2 Methodology

Given the importance of this study, we employ three statistical tests with slightly different properties and strengths to investigate the relationship between VA and HFVDs in CVI thoroughly. Specifically, we employ: (1) Fisher’s exact test (FET); (2) d-variable Hilbert–Schmidt independence criterion (dHSIC) (Pfister et al., 2018); and (3) Kendall’s rank correlation (Tau-c), each of which is described next.

(1) FET is a widely used and well-understood test, but requires us to convert the VA values and HVFD scores to categorical data. The VA values are mapped to five-category data using the vision criteria established by the World Health Organization (2019) (see Table 3).

For HVFDs, the Severity Index is employed for this analysis. This index is mapped to *low* or *high* severity category, depending on whether the index value is below or above the average severity value.

The VA and HVFD categories together create a 2 × 5 contingency matrix (two categories of HVFD severity and five categories of VA levels), where each participant is assigned to one of the 10 cells in the matrix. Table 4 presents this contingency matrix for our data.

**Table 4:** 2×5 Contingency matrix for Fisher’s exact test on HVFD severity (rows) vs VA category (columns).

	Visual acuity category (World Health Organization, 2019)					
HVFD severity	Normal	Mild	Moderate	Severe	Blind	Totals
Low	26	4	5	1	1	37
High	26	9	9	2	0	45
Totals	52	13	14	3	1	83

The null hypothesis for the Fisher’s test is that the two populations, participants with *low* HVFD severity, and participants with *high* HVFD severity are equally likely to have different VA levels. Thus, the alternative hypothesis is that the two populations are not equally likely to have different VA levels.

(2) The second statistical test that we employ to investigate the relationship between VA and HVFDs is a more recent and rigorous test that eliminates the need to convert the VA and HVFD scores to categorical data. dHSIC directly determines dependence between two vectors: the VA value and HVFD Severity Index without the need to choose thresholds to create a contingency matrix, thus avoiding the loss of information that occurs due to thresholding or categorizing. The dHSIC test poses the null hypothesis that all variables are jointly independent. (In our setup, since we are testing only two variables, VA and HVFD Severity, joint independence is equivalent to pairwise independence.) The dHSIC test returns two key numbers: *test statistic* and *critical value*. The null hypothesis is rejected if the *test statistic* is strictly greater than *critical value* (that is, the two values cannot be equal).
Finally, the third test, Kendall’s rank correlation is used to test (1) the directionality of the relationship (i.e., positive, inverse, no correlation) and (2) the strength of the relationship (i.e., perfect, moderate, or weak correlation) between two ordinal variables such as the LogMAR VA values and the HVFD severity scores. In general, Kendall’s correlation coefficient (tau) is 1 (highest) when the rankings of the two variables have perfect agreement, and −1 (lowest) when the rankings of the two variables have perfect disagreement. If the two variables are independent, then Kendall’s coefficient is zero.

#### 3.2.3 Results

(1) The Fisher’s exact test on our data gives a *p*-value of 0.202, that is, it fails to reject the null hypothesis. Loosely speaking, this test result suggests that the two populations, *low* HVFD severity and *high* HVFD severity, are equally likely to have different VA levels. In other words, the data does not exhibit any relation between the severity of higher visual function deficits and the VA levels.
(2) The results of the dHSIC test on our data are the *test statistic* is 0.306 and the *critical value* is 0.594. Since the test results show that the *test statistic* is not strictly greater than *critical value*, the dHSIC test fails to reject the null hypothesis for our data. That is, it suggests retaining the null hypothesis—the VA and HVFDs severity are jointly independent.
(3) The Kendall’s rank correlation test on our data gives the Kendall coefficient (tau) of −0.099, demonstrating a clear lack of correlation between VA levels and HVFD severity. In fact, the near-zero tau value suggests that the two variables are independent. The corresponding *p*-value returned by the test (*p*-value = 0.217) is too large to reject the null hypothesis, thus retaining the null hypothesis that VA levels and HVFD severity are independent.

**Figure 7:**
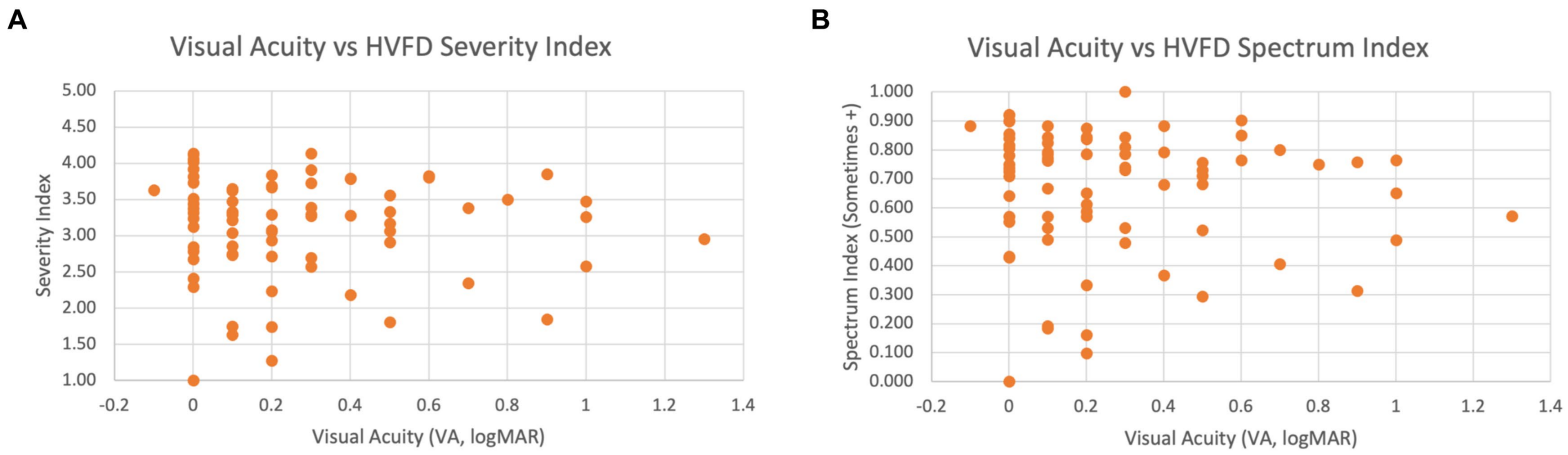
Scatter plot of VA versus HVFD for children with CVI (*n* = 83). (A) VA vs. HVFD Severity Index. (B) VA vs. HVFD Spectrum Index.

Figure 7A shows a scatterplot of the VA and the HVFD Severity Index, which visually illustrates a general lack of correlation between the two factors. Figure 7B compares the HVFD Spectrum Index with VA values, and similar trends are evident with this metric as well.

Overall, we see a clear consensus between all three tests in demonstrating that the wide spectrum of HVFDs elicited by the HVFQI are not independent of VA.

### 3.3 Objective 3: study the relationship between age and HVFDs in children with CVI

The relationship between HVFDs and age has not yet been broadly characterized. Previous studies suggest that visual function improves with age among children with CVI due to physiological visual processing development and neuroplasticity (Bennett et al., 2020; Galli et al., 2022; Watson et al., 2007; Matsuba and Jan, 2006; Lim et al., 2005). This indicates that there is a negative correlation between age and relative visual function deficit severity, but HVFs were not studied. We doubt if there is a relationship between VA and HVFDs with varying age and attempt an initial investigation of age and absolute severity and spectrum of HVFDs in our cross-sectional cohort and suggest further studies.

For this investigation, we employ the same two statistical tests that we employed earlier: (i) dHSIC to test for independence between age and HVFDs and (ii) Kendall’s rank correlation to determine if there is an ordinal association between HVFDs and age. We have to omit Fisher’s exact test because it requires categorical data and there are no conventions for mapping the 4- to 18-year age range to categories.

#### 3.3.1 Data

Ninety two children with established diagnosis of CVI (group CVI).

#### 3.3.2 Results

The results from this preliminary analysis are as follows:

1) For the dHSIC test, the null hypothesis is that age and HVFD Severity are jointly independent. The results from the dHSIC test are as follows: *test statistic* = 0.791; *critical value* = 0.560. Since the *test statistic* is strictly greater than the *critical value*, the dHSIC test rejects the null hypothesis. Thus, age and HVFD severity are not independent as per the dHSIC test.
2) The Kendall’s rank correlation test provides a *tau* value of 0.123, which suggests a weak positive correlation between age and HVFD severity. However, the corresponding *p-value* is 0.102, which is too high and in the context of correlation analysis indicates that the observed correlation between age and HVFD severity is by chance, that is, the correlation is really 0.

These contradictory results underscore the preliminary nature of this investigation. A larger study—longitudinal and controls for factors such as the onset age, developmental environments, and habilitations—is part of our near-term future study plan.

**Figure 8:**
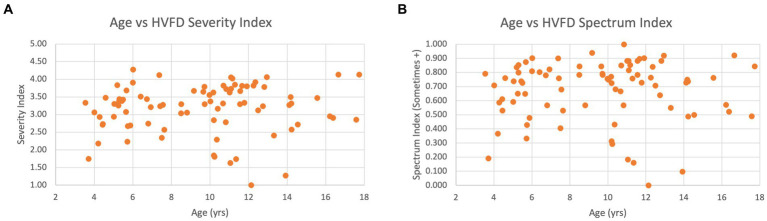
Scatter plot of age versus HVFD for children with CVI (*n* = 92). (A) Age vs. HVFD Severity Index. (B) Age vs. HVFD Spectrum Index.

Figure 8A shows a scatterplot of age and HVFD severity index, which visually illustrates a general lack of correlation between the two factors. Figure 8B compares HVFD Spectrum Index with age, and similar trends are evident with this metric as well.

Overall, the analysis hints at a relationship between participant’s age and their higher visual function deficits; however, larger studies need to be undertaken to conclusively establish the strength and polarity of this relationship.

## 4 Discussion

Our overarching goal behind the three-pronged investigation, described in the prior sections, is to deepen our understanding of the higher visual function deficits in children with CVI and the relationship between HVFDs and visual acuity. Overall, our findings and those of previous studies examining VA and HVFDs (van Genderen et al., 2012; Chandna et al., 2021) provide substantial evidence to support the integration of HVFQI into clinical and habilitational practice for children with CVI regardless of VA measures, where applicable.

### 4.1 The HVFQI is effective in eliciting HVFDs in children with CVI

Our first significant finding confirms and adds to our previous research of the ability of HVFQI to elicit HVFDs, showing Likert scale scores that are clearly distinct (higher) from those of neurotypical participants (Figure 4). This is important for establishing the HVFQI as an effective clinical tool. That being said, the instrument is not perfect and the following discussion on error analysis and possible contributing factors provide further insights for future improvement.

As seen in Figure 1, both the participant groups have outliers—there are seven participants in the NT group for whom the HVFD Severity Index is higher than the expected variation in NT group’s distribution (blue dots), and five participants in the CVI group with Severity Index lower than the expected variation in CVI group’s distribution (orange dots; 4 overlapping at 0.0).

We suspect that the seven outliers in the NT group are participants with false-negative cases with HVFD traits suggestive of CVI. The basis for our suspicion is the result from a recent study by Williams et al. (2021) where they observed that one in 30 children in the primary school population have CVI traits of HVFDs presumably from undiagnosed CVI. This result confirms their recommendation for further investigation into the problem of underdiagnosis of CVI among children.

The five outliers in the CVI group with scores typical of NT group present a more complicated scenario because there are several possible factors at play here:

1. Participants incorrectly answered the questions. Two of the participants with CVI diagnosis chose *Never* for all the questions. In our study, the interviewer asked the question but refrained from influencing the response. Survey fatigue might also be a contributing factor (Gibson and Bowling, 2019; Tourangeau et al., 2010).
2. Participants have such severe developmental disabilities that they may be incorrectly diagnosed as CVI (false-positive CVI cases). Four participants out of the five outliers had a history of significant brain-based comorbidity conditions. The fifth child was one of the triplets with very low birth weight and had suffered traumatic brain injury (classified as mild). Prematurity and low birth weight have been associated with CVI behaviorisms (Dutton, 2013; Macintyre-Béon et al., 2013; Slidsborg et al., 2012; Geldof et al., 2015) as is mild traumatic brain injury (Rauchman et al., 2022; Brosseau-Lachaine et al., 2008). However, this may be because of other masking comorbidities and severe developmental disabilities (Jimenez-Gomez et al., 2022).
3. Participants have adapted to HVFDs. Children often develop efficient adaptations to compensate for deficits of various nature including ocular, which in the current scenario can mask the observation of HVFDs (Zihl and Dutton, 2015; Ortibus et al., 2019).

These observations about the outliers suggest further research into questionnaire administration methodology and into the development of shorter questionnaire and screeners (Chandna et al., 2021). Studies to develop tools and tests that can better distinguish CVI traits from common comorbidities are also needed. Finally, NT outliers found to have HVFD traits on the HVFQI may need further assessment to determine whether they have CVI. The outliers in the CVI group should have further assessments, for example, to determine whether they have developed adaptations that mask the underlying HVFDs.

While this study confirms the efficacy of the HVFQI for quantitative characterization of HVFDs as demonstrated in Chandna et al. (2021), it goes beyond just that finding. We add quantitative measures of the spectrum and severity index for each participant.

#### 4.1.1 HVFD severity index and spectrum index

The ability of HVFQI to capture and quantify both the spectrum and the severity of HVFDs for each child is powerful. For clinicians, the indices and the information about HVFD spectrum and severity are crucial for the following reasons:

(1) These measures help understand HVFDs by viewing a 2-D picture as they affect the individual child. For example, a child with CVI may have a narrow spectrum in the presence of high severity distinct from another child with a broad spectrum with low severity. Considered together, this 2-D view would need a different (re)habilitational approach for the two children.

(2) Longitudinal measures of spectrum and severity scores and the changes in the Likert response scale for the HVFDs would help monitor the efficacy of intervention and, if necessary, indicate a modification of strategies.

(3) These measures would provide practical information to the family, school, and child and response to therapy.

### 4.2 HVFDs are independent of VA loss

Our third significant finding from this investigation, taken together with our previous results, provides conclusive evidence for the independence of HVFDs from visual acuity measures. Previous study had studied the effectiveness of HVFQI only for children with normal VA. Our findings substantially expand the applicability of the HVFQI for identifying HVFDs in children with reduced levels of VA (normal to 1.0 Log MAR VA) for both spectrum and severity of HVFD. This finding should enable us to make substantial progress toward including HVFD characterization in the following: (a) a more comprehensive definition of CVI; (b) emerging diagnostic criteria; (c) the spectrum and severity in the description of CVI; and (d) International Classification of Diseases (ICD) coding for the description of CVI (to accurately reflect the criteria for the definition of CVI). The independence of HVFDs from VA measures also means that for children and families, (re)habilitational measures should include accommodation and intervention strategies that are aimed toward HVFDs, including a multisensory approach and not entirely dependent on the strategies for improvements in visual acuity.

What possible model could explain this independence of higher visual function deficits from visual acuity? A possible reason for this independence may lie in the structural and functional organization of the visual pathways and their function. Neural pathways for vision can be anatomically identified from their origin in retinal ganglion cells (RGCs) in the eye. Evidence from vertebrate studies has identified several classes of RGCs across the retina with specific visual functions. For example, a population of RGCs conveys features of the visual scene and another population the spatial resolution of features (Sanes and Masland, 2015). In addition, the details of visual information transmitted from the retina are dependent on several factors of which two are important. First, the density of RGCs and their convergence with photoreceptors are interrelated (Dacey, 1993). The higher density and lower convergence ratio (almost 1:1) of RGCs in the central foveal area are responsible for high spatial resolution, as required for VA. Conversely, lower density and higher convergence ratios in the peripheral retina result in a lower spatial resolution but still convey important information from the peripheral visual field, enabling enhanced motion detection and spatial information without conscious awareness or “scene gist recognition” (Oliva and Torralba, 2006; Larson and Loschky, 2009). This separation of visual information continues through the optic nerve: the multilayered dorsal lateral geniculate nucleus (dLGN) (Dhande and Huberman, 2014) and specific visual areas *beyond* the primary visual cortex (area V1) (Robles et al., 2014; Nealey and Maunsell, 1994). In addition, magnocellular fibers largely originate from the peripheral RGCs and go to the parietal lobe (dorsal stream). The parvocellular fibers largely originate from the central RGC conveying information from the cone-rich fovea to the infero-temporal cortex (ventral stream) (Livingstone and Hubel, 1987; Nealey and Maunsell, 1994; Maunsell, 1987; Milner and Goodale, 1993). Further information of this dichotomy is provided by lesion studies of the primate visual system. Lesions in the magnocellular pathway in macaques affected the motion responses in the middle temporal area (Maunsell et al., 1990) stationed in the dorsal stream whereas a similar lesion in the parvocellular pathway did not. Lesioning of the parvocellular cells in the LGN of the macaque led to a three-fold reduction in visual acuity, whereas magnocellular lesion in the LGN did not affect visual acuity (Merigan et al., 1991). In humans, a study of the acquired pathology in the medial dorsal visual stream revealed impairment of spatial perception, judgment of symmetry, and visual search with normal perceptual identification of objects and letters (Castelo-Branco et al., 2006). Recent evidence from structure and function studies with conventional magnetic resonance imaging (MRI), fiber tracking of optic radiation, the ganglion cell layer, and visual field assessment show loss of retinal ganglion cells (Lennartsson et al., 2014; Lennartsson et al., 2021) likely through retrograde trans-synaptic degeneration (Jindahra et al., 2009; Dinkin, 2017; Jindahra et al., 2012), lending credence to this model. In adults with CVI where the ganglion cell layer in the central area was preserved with sparing of the central visual field, VA in that eye was normal in the presence of significant dorsal stream deficits (Lennartsson et al., 2021). Based on these studies and our results, a possible model explaining the independence of HVFDs from VA is emerging, which will need further investigation.

### 4.3 HVFD challenges are present at all ages within our study age group

Our preliminary analysis with two different statistical tests showed contradictory results for the relationship between HVFD and age. This was not our primary objective but as an exploration; it highlights the need for further studies of this important relationship. The natural history of HVFDs may show declining challenges of HVFDs with age, indicating improvement with visual development. The opposite may be true. In addition, new HVFD challenges may appear due to increased interaction with the community and the complexity of tasks required. The impact of early diagnosis, introduction of accommodation strategies, and targeted interventions for the spectrum and severity of HVFDs also need to be studied. The results of such studies will help guide therapy, provide information to parents of children with CVI, older children, and provide insight into the neural mechanisms underlying HVFDs in CVI and potential neuroplasticity. Longitudinal studies of the spectrum and severity of HVFDs with clinical and treatment information are necessary.

### 4.4 HVFQI as a clinical tool

This study confirms the efficacy of the HVFQI as a clinical tool to elicit HVFDs and to provide a comprehensive assessment of HFVs. Our two prospective controlled studies in children with confirmed diagnoses of CVI have provided the evidence. Our findings are similar to earlier studies with similar questionnaires and largely retrospective analysis but without a control population (Macintyre-Béon et al., 2012; van Genderen et al., 2012; Duke et al., 2021). We have also added to the previous versions of the original Insight and CVI-I inventories by (a) improving the set of questions, (b) including the *Not Applicable* response in analysis; and (c) converting from paper-based to a web-based modality that is interactive and scalable and thus universally accessible. Adding the spectrum and severity indices and also the probability of the diagnosis of HVFDs in CVI using machine learning models have increased the informational value that the HVFQI app provides to the clinicians and consequentially its adoption in clinical practice.

### 4.5 Limitations of our study

On average, the time required to administer the complete HVFQI (51 questions) to a participant is 31 ± 14 min (mean ± SD). Although that is a justifiable duration given the breadth and depth of the information that is gathered during that session, the time commitment can become onerous for time-pressed clinicians, teachers, and busy families. To address this concern, we are developing a self-administration functionality for the HVFQI, and guidance is provided to the respondent along with the ability to submit an assistance request for clarification on specific questions. We expect this functionality to help clinicians make efficient use of their time and allow families the flexibility to respond to the HVFQI from any location, any time, and over multiple sittings within a prescribed time window. We are also further validating the Top-11Q Screener (Chandna et al., 2021) comprising 11 questions in a separate controlled study in a wider cohort and to tackle time restraints and improve the applicability of the HVFQI to large populations.

The questions in the current version of the HVFQI are designed for 4- to 18-year-old children. However, for children with severe visual impairment, some of the questions may be inapplicable as the observation of visually guided behaviors by parents can be challenging. Similarly, children with comorbidities may have physical and/or cognitive limitations that prevent them from engaging in certain activities and thus rendering many of the questions on the HVFQI inapplicable, for example, “Does your child have difficulty walking downstairs?”. For now, we have the *Not Applicable* option with free text for this reason, which is part of our data and analysis. In the future, the analysis of the questions answered as not applicable and correlation with comorbidities will help us develop new questions or modify the present inventory to a more appropriate inventory specifically for these populations.

## 5 Conclusion

In conclusion, this study underscores the independence of higher visual function deficits (HVFDs) from visual acuity (VA) in children with cerebral visual impairment (CVI), highlighting the necessity of incorporating the Higher Visual Function Question Inventory (HVFQI) into clinical practice. Our findings support a more comprehensive definition of CVI that includes HVFDs, urging revisions to diagnostic criteria and ICD coding. The HVFQI reliably distinguishes between neurotypical children and those with CVI, providing a valuable spectrum and severity indices for individualized intervention. The development of a web-based HVFQI and validation of a shorter screener will enhance its clinical utility. Despite the time commitment required, future modifications will ensure its applicability across diverse populations. All children with suspected CVI should be assessed for HVFDs alongside VA measures to improve the understanding and treatment of CVI.

On a broader level, the insights gleaned through the HVFQI responses of cohorts of children will increase our understanding of the natural history of CVI and its progression by affording us opportunities to study CVI and its relationship with the common etiologies and brain imaging findings, while also evaluating the impact of comorbidities such as cerebral palsy. This will aid in developing conceptual models of HVFDs in CVI. Of even greater significance is the potential of employing the HVFQI for longitudinal studies that inform the natural history of HVFDs in CVI, (re)habilitation measures, monitor their efficacy, and guide their adaptation.

## Data Availability

The raw data supporting the conclusions of this article will be made available by the authors, without undue reservation.

## Footnotes

^1^We use Neurotypical (NT), and Typical, interchangeably throughout the paper including in figures.

^2^Chandna et al. (2021) refer to this metric as *Average Score.*

^3^Chandna et al. (2021) mapped the categorical response labels to 0–4 range. In this work we have chosen to use the typical Likert scale mapping of 1–5.

^4^A boxplot or box-and-whisker plot reports five-point summary of the data distribution: minimum, first quartile, second quartile (median), third quartile, and maximum.

^5^The RRF@Sometimes+ metric is equivalent to the HVFD Spectrum Index.

^6^Q7. Does your child leave food on the near or far side of their plate? Q8. Does your child leave food on the right or left side of their plate? Q23. Does your child get lost in places which are well known to them?

^7^Q1. Does your child trip over toys and obstacles on the floor? Q2. Does your child have difficulty walking down the stairs? Q49. Does your child have difficulty naming common colours?

## References

[ref1] Bauer C. M. Merabet L. B. (2024). Aberrant white matter development in cerebral visual impairment: a proposed mechanism for visual dysfunction following early brain injury. J. Integr. Neurosci. 23:1. doi: 10.31083/j.jin2301001, PMID: 38287851 PMC12434583

[ref2] Bennett C. R. Bauer C. M. Bailin E. S. Merabet L. B. (2020). Neuroplasticity in cerebral visual impairment (CVI): assessing functional vision and the neurophysiological correlates of dorsal stream dysfunction. Neurosci. Biobehav. Rev. 108, 171–181. doi: 10.1016/j.neubiorev.2019.10.011, PMID: 31655075 PMC6949360

[ref3] Bennett C. R. Bex P. J. Bauer C. M. Merabet L. B. (2019). The assessment of visual function and functional vision. Semin. Pediatr. Neurol. 31, 30–40. doi: 10.1016/j.spen.2019.05.006, PMID: 31548022 PMC6761988

[ref4] Bosch D. G. Boonstra F. N. Willemsen M. A. Cremers F. P. Vries B. (2014). Low vision due to cerebral visual impairment: differentiating between acquired and genetic causes. BMC Ophthalmol. 14, 1–9. doi: 10.1186/1471-2415-14-59 24886270 PMC4021540

[ref6] Brosseau-Lachaine O. Gagnon-I. Forget R. Faubert J. (2008). Mild traumatic brain injury induces prolonged visual processing deficits in children. Brain Inj. 22, 657–668. doi: 10.1080/02699050802203353, PMID: 18698516

[ref7] Castelo-Branco M. Mendes M. Silva M. F. Januário C. Machado E. Pinto A.. (2006). Specific retinotopically based magnocellular impairment in a patient with medial visual dorsal stream damage. Neuropsychologia 44, 238–253. doi: 10.1016/j.neuropsychologia.2005.05.005, PMID: 16005479

[ref8] Chandna A. Ghahghaei S. Foster S. Kumar R. (2021). Higher visual function deficits in children with cerebral visual impairment and good visual acuity. Front. Hum. Neurosci. 15:711873. doi: 10.3389/fnhum.2021.711873, PMID: 34867236 PMC8636735

[ref9] Chang M. Y. Borchert M. S. (2021). Validity and reliability of eye tracking for visual acuity assessment in children with cortical visual impairment. J. AAPOS 25, 334.e1–334.e5. doi: 10.1016/j.jaapos.2021.07.008 34687876

[ref10] Chokron S. Dutton G. N. (2016). Impact of cerebral visual impairments on motor skills: implications for developmental coordination disorders. Front. Psychol. 7:1471. doi: 10.3389/fpsyg.2016.01471, PMID: 27757087 PMC5048540

[ref11] Colenbrander A. (2003). Aspects of vision loss – visual functions and functional vision. Vis. Impair. Res. 5, 115–136. doi: 10.1080/1388235039048919

[ref12] Colenbrander A. (2010a). What’s in a name? Appropriate terminology for CVI. J. Vis. Impair. Blind 104, 583–585. doi: 10.1177/0145482X1010401002

[ref13] Colenbrander A. (2010b). “Towards the development of a classification of vision- related functioning- a potential framework” in Clinics in developmental medicine. eds. Dutton G. N. Bax M. (London: MacKeith Press).

[ref15] Dacey M. D. (1993). The mosaic of midget ganglion cells in the human retina. J. Neurosci. 13, 5334–5355. doi: 10.1523/JNEUROSCI.13-12-05334.1993, PMID: 8254378 PMC6576399

[ref16] Dhande O. S. Huberman A. D. (2014). Retinal ganglion cell maps in the brain: implications for visual processing. Curr. Opin. Neurobiol. 24, 133–142. doi: 10.1016/j.conb.2013.08.006, PMID: 24492089 PMC4086677

[ref17] Dinkin M. (2017). Trans-synaptic retrograde degeneration in the human visual system: slow, silent, and real. Curr. Neurol. Neurosci. Rep. 17:16. doi: 10.1007/s11910-017-0725-2, PMID: 28229400

[ref18] Duke R. E. Chimaeze T. Kim M. J. Ameh S. Burton K. Bowman R. (2021). The effect of insight questions inventory and visual support strategies on carer-reported quality of life for children with cerebral palsy and perceptual visual dysfunction in Nigeria: a randomized controlled trial. Front. Hum. Neurosci. 15:706550. doi: 10.3389/fnhum.2021.706550, PMID: 34867233 PMC8636698

[ref20] Dutton G. N. (2013). The spectrum of cerebral visual impairment as a sequel to premature birth: an overview. Doc. Ophthalmol. 127, 69–78. doi: 10.1007/s10633-013-9382-1, PMID: 23657712

[ref21] Dutton G. Bax M. (Eds.) (2010). Visual impairment in children due to damage to the brain. Hoboken, USA: John Wiley & Sons.

[ref22] Dutton G. N. Calvert J. Ibrahim H. Macdonald E. McCulloch D. L. Macintyre-Béon C. (2010). “Structured clinical history taking for cognitive and perceptual visual dysfunction and for profound visual disabilities due to damage to the brain in children” in Visual impairment in children due to damage to the brain. eds. Dutton G. D. Bax M. (London: Mac Keith Press).

[ref23] Dutton G. N. Jacobson L. K. (2001). Cerebral visual impairment in children. Semin. Neonatol. 6, 477–485. doi: 10.1053/siny.2001.0078 12014888

[ref24] Dutton G. N. Saaed A. Fahad B. Fraser R. McDaid G. McDade J.. (2004). Association of binocular lower visual field impairment, impaired simultaneous perception, disordered visually guided motion and inaccurate saccades in children with cerebral visual dysfunction—a retrospective observational study. Eye 18, 27–34. doi: 10.1038/sj.eye.6700541, PMID: 14707961

[ref27] Galli J. Loi E. Molinaro A. Calza S. Franzoni A. Micheletti S.. (2022). Age-related effects on the Spectrum of cerebral visual impairment in children with cerebral palsy. Front. Hum. Neurosci. 16:750464. doi: 10.3389/fnhum.2022.750464, PMID: 35308614 PMC8924515

[ref28] Gallivan J. P. Goodale M. A. (2018). The dorsal “action” pathway. Handb. Clin. Neurol. 151, 449–466. doi: 10.1016/B978-0-444-63622-5.00023-1 29519474

[ref29] Geldof C. Wassenaer-Leemhuis A. Dik M. (2015). A functional approach to cerebral visual impairments in very preterm/very-low-birth-weight children. Pediatr. Res. 78, 190–197. doi: 10.1038/pr.2015.83, PMID: 25927544

[ref31] Gibson A. M. Bowling N. A. (2019). The effects of questionnaire length and behavioral consequences on careless responding. Eur. J. Psychol. Assess. 36, 410–420. doi: 10.1027/1015-5759/a000526

[ref32] Goodale M. A. Króliczak G. Westwood D. A. (2005). Dual routes to action: contributions of the dorsal and ventral streams to adaptive behavior. Prog. Brain Res. 149, 269–283. doi: 10.1016/S0079-6123(05)49019-6, PMID: 16226590

[ref33] Hoyt C. S. (2003). Visual function in the brain damaged child. Eye 17, 369–384. doi: 10.1038/sj.eye.6700364, PMID: 12724701

[ref35] Itzhak N. B. Stijnen L. Ortibus E. (2023). The relation between visual orienting functions, visual perception, and functional vision in children with (suspected) cerebral visual impairment. Res. Dev. Disabil. 142:104619. doi: 10.1016/j.ridd.2023.104619 34619456

[ref36] Jacobsen L. (2014). Cerebral dysfunction in children: should this be the central tenet for a new system of classification? Dev. Med. Child Neurol. 56, 101–102. doi: 10.1111/dmcn:12328 24219559

[ref37] Jimenez-Gomez A. Fisher K. S. Zhang K. X. Liu C. Sun Q. Shah V. S. (2022). Longitudinal neurological analysis of moderate and severe pediatric cerebral visual impairment. Front. Hum. Neurosci. 16:772353. doi: 10.3389/fnhum.2022.772353, PMID: 36051970 PMC9425457

[ref38] Jindahra P. Petrie A. Plant G. T. (2009). Retrograde trans-synaptic retinal ganglion cell loss identified by optical coherence tomography. Brain 132, 628–634. doi: 10.1093/brain/awp001, PMID: 19224900

[ref39] Jindahra P. Petrie A. Plant G. T. (2012). The time course of retrograde trans-synaptic degeneration following occipital lobe damage in humans. Brain 135, 534–541. doi: 10.1093/brain/awr324, PMID: 22300877

[ref40] Kran B. S. Lawrence L. Mayer D. L. Heidary G. (2019). Cerebral/cortical visual impairment: a need to reassess current definitions of visual impairment and blindness. Semin. Pediatr. Neurol. 31, 25–29. doi: 10.1016/j.spen.2019.05.005, PMID: 31548020

[ref41] Larson A. M. Loschky L. C. (2009). The contributions of central versus peripheral vision to scene gist recognition. J. Vis. 9:6.1-16. doi: 10.1167/9.10.6, PMID: 19810787

[ref42] Lennartsson F. Nilsson M. Flodmark O. Jacobson L. (2014). Damage to the immature optic radiation causes severe reduction of the retinal nerve fiber layer, resulting in predictable visual field defects. Invest. Ophthalmol. Vis. Sci. 55, 8278–8288. doi: 10.1167/iovs.14-14913, PMID: 25377222

[ref43] Lennartsson F. Öhnell H. M. L. J. Nilsson M. (2021). Pre- and postnatal damage to the retro-geniculate visual pathways cause retinal degeneration predictive for visual function. Front. Hum. Neurosci. 15:734193. doi: 10.3389/fnhum.2021.734193, PMID: 34764861 PMC8577566

[ref44] Lim M. Soul J. S. Hansen R. M. Mayer D. L. Moskowitz A. Fulton A. B. (2005). Development of visual acuity in children with cerebral visual impairment. Arch. Ophthalmol. 123, 1215–1220. doi: 10.1001/archopht.123.9.1215 16157801

[ref45] Livingstone M. S. Hubel D. H. (1987). Psychophysical evidence for separate channels for the perception of form, color, movement, and depth. J. Neurosci. 7, 3416–3468. doi: 10.1523/JNEUROSCI.07-11-03416.1987, PMID: 3316524 PMC6569044

[ref46] Macintyre-Béon C. Ibrahim H. Hay I. Cockburn D. Calvert J. Dutton G. N.. (2010). Dorsal stream dysfunction in children. A review and an approach to diagnosis and management. Curr. Pediatr. Rev. 6, 166–182. doi: 10.2174/157339610793743895

[ref47] Macintyre-Béon C. Young D. Calvert J. Ibrahim H. Dutton G. N. Bowman R. (2012). Reliability of a question inventory for structured history taking in children with cerebral visual impairment. Eye 26, –1393. doi: 10.1038/eye.2012.154, PMID: 22863818 PMC3470063

[ref48] Macintyre-Béon C. Young D. Dutton G. N. (2013). Cerebral visual dysfunction in prematurely born children attending mainstream school. Doc. Ophthalmol. 127, 89–102. doi: 10.1007/s10633-013-9405-y, PMID: 23996515

[ref49] Marquis D. G. (1934). Effects of removal of the visual cortex in mammals, with observations on the retention of light discrimination in dogs. Res. Publ. Assoc. Res. Nerv. Ment. Dis. 13, 558–592.

[ref50] Matsuba C. A. Jan J. E. (2006). Long-term outcome of children with cortical visual impairment. Dev. Med. Child Neurol. 48, 508–512. doi: 10.1017/S0012162206001071 16700945

[ref51] Maunsell J. H. (1987). “Physiological evidence for two visual subsystems” in Matters of intelligence: Conceptual structures in cognitive neuroscience. ed. Vaina L. M. (Netherlands, Dordrecht: Springer), 59–87.

[ref52] Maunsell J. H. Nealey T. A. DePriest D. D. (1990). Magnocellular and parvocellular contributions to responses in the middle temporal visual area (MT) of the macaque monkey. J. Neurosci. 10, 3323–3334. doi: 10.1523/JNEUROSCI.10-10-03323.1990, PMID: 2213142 PMC6570195

[ref53] McConnell E. L. Saunders K. J. Little J. A. (2021). What assessments are currently used to investigate and diagnose cerebral visual impairment (CVI) in children? A systematic review. Ophthalmic Physiol. Opt. 41, 224–244. doi: 10.1111/opo.12776, PMID: 33368471 PMC8048590

[ref54] Merabet L. B. Mayer D. L. Bauer C. M. Wright D. Kran B. S. (2017). Disentangling how the brain is “wired” in cortical (cerebral) visual impairment. Semin. Pediatr. Neurol. 24, 83–91. doi: 10.1016/j.spen.2017.04.005 28941531 PMC5659284

[ref55] Merigan W. H. Katz L. M. Maunsell J. H. (1991). The effects of parvocellular lateral geniculate lesions on the acuity and contrast sensitivity of macaque monkeys. J. Neurosci. 11, 994–1001. doi: 10.1523/JNEUROSCI.11-04-00994.1991, PMID: 2010820 PMC6575382

[ref56] Milner A. D. (2017). How do the two visual streams interact with each other? Exp. Brain Res. 235, 1297–1308. doi: 10.1007/s00221-017-4917-4, PMID: 28255843 PMC5380689

[ref57] Milner A. D. Goodale M. A. (1993). Visual pathways to perception and action. Prog. Brain Res. 95, 317–337. doi: 10.1016/S0079-6123(08)60379-9 8493342

[ref58] Morelli F. Aprile G. Martolini C. Ballante E. Olivier L. Ercolino E.. (2022). Visual function and neuropsychological profile in children with cerebral visual impairment. Children 9:921. doi: 10.3390/children9060921, PMID: 35740858 PMC9221908

[ref59] Nealey T. A. Maunsell J. H. (1994). Magnocellular and parvocellular contributions to the responses of neurons in macaque striate cortex. J. Neurosci. 14, 2069–2079. doi: 10.1523/JNEUROSCI.14-04-02069.1994, PMID: 8158257 PMC6577134

[ref60] Oliva A. Torralba A. (2006). Building the gist of a scene: the role of global image features in recognition. Prog. Brain Res. 155, 23–36. doi: 10.1016/S0079-6123(06)55002-2 17027377

[ref61] Ortibus E. Fazzi E. Dale N. (2019). Cerebral visual impairment and clinical assessment: the European perspective. Semin. Pediatr. Neurol. 31, 15–24. doi: 10.1016/j.spen.2019.05.004, PMID: 31548019

[ref62] Ortibus E. Laenen A. Verhoeven J. Cock P. Casteels I. Schoolmeesters B.. (2011). Screening for cerebral visual impairment: value of a CVI questionnaire. Neuropediatrics 42, 138–147. doi: 10.1055/s-0031-1285908, PMID: 21913154

[ref63] Ortibus E. Verhoeven J. Sunaert S. Casteels I. Cock P. Lagae L. (2012). Integrity of the inferior longitudinal fasciculus and impaired object recognition in children: a diffusion tensor imaging study. Dev. Med. Child Neurol. 54, 38–43. doi: 10.1111/j.1469-8749.2011.04147.x, PMID: 22171928

[ref64] Pfister N. Bühlmann P. Schölkopf B. Peters J. (2018). Kernel-based tests for joint independence. J. R. Stat. Soc. Ser. B Stat. Method. 80, 5–31. doi: 10.1111/rssb.12235

[ref65] Philip S. S. Dutton G. N. (2014). Identifying and characterising cerebral visual impairment in children: a review. Clin. Exp. Optom. 97, 196–208. doi: 10.1111/cxo.12155 24766507

[ref66] Philip S. S. Tsherlinga S. Thomas M. M. Dutton G. N. Bowman R. (2016). A validation of an examination protocol for cerebral visual impairment among children in a clinical population in India. J. Clin. Diagn. Res. 10, NC01–NC04. doi: 10.7860/JCDR/2016/22222.8943, PMID: 28208897 PMC5296470

[ref67] Polanen V. Davare M. (2015). Interactions between dorsal and ventral streams for controlling skilled grasp. Neuropsychologia 79, 186–191. doi: 10.1016/j.neuropsychologia.2015.07.010, PMID: 26169317 PMC4678292

[ref68] Rauchman S. H. Albert J. Pinkhasov A. Reiss A. B. (2022). Mild-to-moderate traumatic brain injury. A review with focus on the visual system. Neurol. Int. 14, 453–470. doi: 10.3390/neurolint14020038, PMID: 35736619 PMC9227114

[ref69] Ravenscroft J. (2016). Where is cerebral visual impairment? Br. J. Vis. Impair. 34, 3–4. doi: 10.1177/0264619615624190

[ref71] Robles E. Laurell E. Baier H. (2014). The retinal projectome reveals brain-area-specific visual representations generated by ganglion cell diversity. Curr. Biol. 24, 2085–2096. doi: 10.1016/j.cub.2014.07.080, PMID: 25155513

[ref72] Rolls E. T. (1991). Neural organization of higher visual functions. Curr. Opin. Neurobiol. 1, 274–278. doi: 10.1016/0959-4388(91)90090-T 1821190

[ref73] Sakki H. E. Dale N. J. Sargent J. Perez-Roche T. Bowman R. (2017). Is there consensus in defining childhood cerebral visual impairment? A systematic review of terminology and definitions. Br. J. Ophthalmol. 102, 424–432. doi: 10.1136/bjophthalmol-2017-310694 29146757

[ref74] Sanes J. R. Masland R. H. (2015). The types of retinal ganglion cells: current status and implications for neuronal classification. Annu. Rev. Neurosci. 38, 221–246. doi: 10.1146/annurev-neuro-071714-034120, PMID: 25897874

[ref75] Sarubbo S. Benedictis A. Maldonado I. L. Basso G. Duffau H. (2013). Frontal terminations for the inferior fronto-occipital fascicle: anatomical dissection, DTI study and functional considerations on a multi-component bundle. Brain Struct. Funct. 218, 21–37. doi: 10.1007/s00429-011-0372-3, PMID: 22200882

[ref77] Slidsborg C. Bangsgaard R. Fledelius H. C. Jensen H. Greisen G. Cour M. (2012). Cerebral damage may be the primary risk factor for visual impairment in preschool children born extremely premature. Arch. Ophthalmol. 130, 1410–1417. doi: 10.1001/archophthalmol.2012.1393, PMID: 22688255

[ref78] Sprague J. M. Meikle T. H. Jr. (1965). The role of the superior colliculus in visually guided behavior. Exp. Neurol. 11, 115–146. doi: 10.1016/0014-4886(65)90026-9 14272555

[ref80] Tourangeau R. Groves R. M. Redline C. D. (2010). Sensitive topics and reluctant respondents: demonstrating a link between non-response bias and measurement error. Public Opin. Q. 74, 413–432. doi: 10.1093/poq/nfq004

[ref81] Vancleef K. Janssens E. Petré Y. Wagemans J. Ortibus E. (2020). Assessment tool for visual perception deficits in cerebral visual impairment: reliability and validity. Dev. Med. Child Neurol. 62, 118–124. doi: 10.1111/dmcn.14304, PMID: 31267523

[ref30] van Genderen M. Dekker M. Pilon F. Bals I. (2012). Diagnosing cerebral visual impairment in children with good visual acuity. Strabismus 20, 78–83. doi: 10.3109/09273972.2012.680232 22612357

[ref83] Watson T. Orel-Bixler D. Haegerstrom-Portnoy G. (2007). Longitudinal quantitative assessment of vision function in children with cortical visual impairment. Optom. Vis. Sci. 84, 471–480. doi: 10.1097/OPX.0b013e31806dba5f, PMID: 17568316

[ref84] Weiller C. Reisert M. Peto I. Hennig J. Makris N. Petrides M.. (2021). The ventral pathway of the human brain: a continuous association tract system. NeuroImage 234:117977. doi: 10.1016/j.neuroimage.2021.117977, PMID: 33757905

[ref85] Whiting S. Jan J. E. Wong P. K. Flodmark O. Farrell K. McCormick A. Q. (1985). Permanent cortical visual impairment in children. Dev. Med. Child Neurol. 27, 730–739. doi: 10.1111/j.1469-8749.1985.tb03796.x 4092845

[ref86] Williams C. Pease A. Warnes P. Harrison S. Pilon F. Hyvarinen L.. (2021). Cerebral visual impairment-related vision problems in primary school children: a cross-sectional survey. Dev. Med. Child Neurol. 63, 683–689. doi: 10.1111/dmcn.14819, PMID: 33533021

[ref87] World Health Organization (2019). World Report on Vision, 2019. Geneva: World Health Organization.

[ref88] Yeatman J. D. Weiner K. S. Pestilli F. Rokem A. Mezer A. Wandell B. A. (2014). The vertical occipital fasciculus: a century of controversy resolved by in vivo measurements. Proc. Natl. Acad. Sci. 111, 5214–5223. doi: 10.1073/pnas.1418503111 PMC4260539 25404310

[ref89] Zihl J. Dutton G. N. (2015). “Visual disorders” in Cerebral visual impairment in children: visuoperceptive and visuocognitive disorders. Ed. K. Lenhart and W. McHugh. Springer., 61–115.

[ref90] Zihl J. Kennard C. (2003). “Disorders of higher visual function” in Neurological disorders: course and treatment. eds. Brandt T. Caplan L. R. Dichgans J. Diener H. C. Kennard C.. 2nd ed, 255–263. doi: 10.1016/B978-012125831-3/50219-7

